# Treatment of metastatic malignant melanoma with dacarbazine, vindesine and cisplatin.

**DOI:** 10.1038/bjc.1989.327

**Published:** 1989-10

**Authors:** D. Pectasides, H. Yianniotis, N. Alevizakos, D. Bafaloukos, V. Barbounis, J. Varthalitis, M. Dimitriadis, A. Athanassiou

**Affiliations:** Department of Medical Oncology, Metaxas Memorial Cancer Hospital, Piraeus, Greece.

## Abstract

Twenty-seven patients with disseminated malignant melanoma were treated monthly with cisplatin (CDDP) 120 mg m-2 on day 1, vindesine (VDS) 3 mg m-2 on day 2 and dacarbazine (DTIC) 250 mg m-2 on days 2-6. None of them had received prior chemotherapy. All patients are evaluable for response and toxicity. There were five (19%) complete (CR) and seven (26%) partial (PR) responses for a total response rate of 45%. We conclude that the combination of DTIC, VDS and CDDP is capable of producing a relatively high rate of response in patients with advanced metastatic malignant melanoma, but responses are short.


Br. J. Cancer (1989), 60, 627-629 © The Macmillan Press Ltd., 1989

### Treatment of metastatic malignant melanoma with dacarbazine, vindesine and cisplatin

D. Pectasides, H. Yianniotis, N. Alevizakos, D. Bafaloukos, V. Barbounis, J. Varthalitis, M. Dimitriadis & A. Athanassiou

*First Department of Medical Oncology, The Metaxas Memorial Cancer Hospital, 51 Botassi Street, Piraeus 18537, Greece.*

**Summary** Twenty-seven patients with disseminated malignant melanoma were treated monthly with cisplatin (CDDP) 120 mg $m^{-2}$ on day 1, vindesine (VDS) 3 mg $m^{-2}$ on day 2 and dacarbazine (DTIC) 250 mg $m^{-2}$ on days 2-6. None of them had received prior chemotherapy. All patients are evaluable for response and toxicity. There were five (19%) complete (CR) and seven (26%) partial (PR) responses for a total response rate of 45%. We conclude that the combination of DTIC, VDS and CDDP is capable of producing a relatively high rate of response in patients with advanced metastatic malignant melanoma, but responses are short.

There is documented activity of vindesine (VDS) (Retsas *et al.*, 1980) and dacarbazine (DTIC) (Comis, 1976; Constanzi, 1976; Pritchard *et al.*, 1981) as single agents or in combination in malignant melanoma with objective response rates of 15-42% (Einhorn *et al.*, 1974; Hill *et al.*, 1984; Retsas *et al.*, 1985). Numerous attempts have been made to increase the response rate by combining various drugs. However, there is no evidence that any particular combination has improved these results (Einhorn *et al.*, 1974; Karakousis *et al.*, 1979). Cisplatin (CDDP) alone or in combination with other active agents has been tried in patients with advanced malignant melanoma and has resulted in a wide range of remission rates (Chary *et al.*, 1977; Nathanson *et al.*, 1981; Al-Sarraf *et al.*, 1982; York *et al.*, 1983; Retsas *et al.*, 1985; Wussow *et al.*, 1987).

Since DTIC, VDS and CDDP have different mode of action and toxicity, it was rational to combine these drugs in an effort to improve response rates and possibly response duration.

### Patients and methods

From March 1984 to December 1986, 27 consecutive patients entered the trial. Entry criteria included histologically documented melanoma, age < 75 years, ECOG performance status of 0-2, predicted survival > 2 months, no prior chemotherapy and creatinine clearance > 60 ml $min^{-1}$.

Chemotherapy consisted of CDDP 120 mg $m^{-2}$ as a 30 min infusion in 3% hypertonic saline with hydration and mannitol diuresis on day 1, VDS 3 mg $m^{-2}$ (no more than 5 mg) on day 2 as an i.v. bolus and DTIC 250 mg $m^{-2}$ on days 2-6 as an i.v. infusion over 30 min. The course was repeated every 4 weeks and doses were decreased according to the degree of haematological and renal toxicity of the preceding course.

Antiemetic treatment with metoclopramide 2 mg $kg^{-1}$ 0.5 h before and 1.5, 3.5, 5.5 and 8 h after CDDP administration plus dexamethasone 8 mg 3 h before and 3, 6 and 9 h after CDDP administration was given. On days 2-6 metoclopramide 2 mg $kg^{-1}$ plus dexamethasone 8 mg 0.5 h before and 3 h after DTIC administration were given.

Fourteen males and 13 females with a mean age of 54 years entered the trial. Patient characteristics are shown in Table I. The mean number of administered courses was four (range 1-8 courses). World Health Organization (WHO) response and toxicity criteria were used (WHO, 1979).

Correspondence: D. Pectasides.
Received 4 October 1988; and in revised form 22 May 1989.

**Table I** Patient characteristics

| *Characteristic* | *No. of patients (%)* |
|---|---|
| Total no. entered | 27 |
| Sex | |
| Male | 14 (52) |
| Female | 13 (48) |
| Age (years) | |
| Mean | 54 |
| Median | 57 |
| Range | 36-72 |
| ECOG performance status | |
| 0 | 6 (22.3) |
| 1 | 11 (40.7) |
| 2 | 10 (37.0) |
| Site of metastases | |
| Subcutaneous and skin | 14 (52) |
| Lymph nodes | 15 (56) |
| Lung | 14 (52) |
| Liver | 1 (4) |
| Bone | 2 (8) |

### Results

### *Response to therapy*

The response to therapy was as follows: complete response (CR) five (19%) patients, partial response (PR) seven (26%), stable (SD) or progressive disease (PD) 15 (55%). The median duration of CR was 4+ months (4,4,4,4.5,9+) and of PR 8 months (3,4.5,5.5,8,10,11,18). Tumour responses by site are shown in Table II. Metastases in the lungs, lymph nodes, subcutaneous tissues and skin were more responsive to chemotherapy. As shown in Table II, CRs were seen in three patients with lung, subcutaneous and lymph node metastases and in two patients with lung and lymph node metastases. The seven patients responding partially to chemotherapy were: one with lung metastases, one with lung and lymph nodes metastases, two with subcutaneous and skin disease, two with lymph nodes metastases and one with lymph node, subcutaneous and skin metastases. There were only four patients with visceral metastatic deposits. Of these there were one PR and three SD or PD. Of the 12 patients with non-visceral disease, five responded partially and seven had SD or PD.

### *Toxicity*

This schedule was well tolerated with moderate toxicity. The toxic effects are summarised in Table III. Three patients developed grade III and IV leucopenia and one of these developed severe lung infection, which was successfully treated with broad spectrum antibiotics. There were no

**Table II** Metastatic sites

| *Response* | *Subcutaneous skin* | *Lymph nodes* | *Lung* | *Subcutaneous skin + bones* | *Lung + lymph nodes* | *Lung + subcutaneous skin* | *Liver + lung* | *Lung + bones* | *Subcutaneous skin + lymph nodes* | *Subcutaneous skin + lymph nodes + lung* | *Total* |
|---|---|---|---|---|---|---|---|---|---|---|---|
| CR | – | – | – | – | 2 | – | – | – | – | 3 | 5 |
| PR | 2 | 2 | 1 | – | 1 | – | – | – | 1 | – | 7 |
| SD or PD | 3 | 2 | 1 | 1 | 2 | 2 | 1 | 1 | 2 | – | 15 |

Only visceral metastatic disease, 4: 1 PR, 3 SD or PD.
Only non-visceral metastatic disease, 12: 5 PR, 7 SD or PD.

**Table III** Toxicity

| | *Grade* | | | | |
|---|---|---|---|---|---|
| | *0* | *I* | *II* | *III* | *IV* |
| Anaemia | 15 | 12 | 0 | 0 | 0 |
| Leucopenia | 9 | 7 | 8 | 2 | 1 |
| Thrombocytopenia | 23 | 3 | 1 | 0 | 0 |
| nausea/vomiting | 0 | 1 | 5 | 12 | 9 |
| Nephrotoxicity | 9 | 12 | 6 | 0 | 0 |
| Neurotoxicity | 25 | 2 | 0 | 0 | 0 |
| Hearing impairement | 27 | 0 | 0 | 0 | 0 |

treatment-related deaths. Despite hydration and mannitol diuresis 18 patients developed transient and reversible nephrotoxicity of grade I and II (increased urea and/or creatinine). Two patients developed peripheral neuropathy, with symptomatic paresthesias and sensory deficit. Nausea and vomiting were nearly universal, but manageable. No patient developed socially noticeable hearing loss.

### Discussion

Although the prognosis of patients with disseminated malignant melanoma remains poor, intensive chemotherapy may produce remissions in a small number of patients. The antitumour effect of VDS in advanced malignant melanoma is at least comparable with, and probably superior to, DTIC (Retsas *et al.*, 1980). It has been reported that the response rate in disseminated malignant melanoma with CDDP alone or in combination with DTIC was no better than the rate obtained with DTIC alone (Friedman *et al.*, 1979; Oratz *et al.*, 1987).

The VBD (vinblastine, bleomycin, cisplatin) combination has been reported to give objective responses ranging from 0% (York *et al.*, 1983) to 43% (Nathanson *et al.*, 1981). Other investigators reported response rates in the intermediate range 10–41% (Creagan *et al.*, 1981; Canadian Melanoma Group, 1984; Luikart *et al.*, 1984; Mechl *et al.*, 1985). Carey *et al.* (1986) reported 25% CR + PR in previously untreated patients with metastatic malignant melanoma by using the VDD (vinblastine, dacarbazine, cisplatin) regime. The (B) $DPV_3$ (Retsas *et al.*, 1985) (cisplatin, vinblastine, vindesine ± bleomycin alternating with dacarbazine, vincristine) regime gave a 29% response rate despite more advanced disease at the onset of treatment. It was concluded that the addition of bleomycin to the $DPV_3$ regime had no effect on response.

The response rate of 45% (CR + PR) for DVP combination in our study is slightly lower than that recently reported by Wussow *et al.* (1987). Toxicity encountered in our patients was similar to those reported by Wussow *et al.* (1987), who used an almost identical regime. The major difference between the previous studies and the present one is that they employed lower doses of cisplatin. Whether the increased dose is important or whether our and Wussow *et al.*'s (1987) better response rate was a chance occurrence cannot be determined. The sites most likely to respond to chemotherapy remain skin, lymph nodes and lungs and no responses were observed in hepatic and skeletal metastases.

The DVP combination as first line chemotherapy in disseminated malignant melanoma seems to yield a useful response rate with moderate toxicity, but the mean duration of response remains disappointing.

### References

AL-SARRAF, M., FLETCHER, W., OISHI, N. & 5 others (1982). Cisplatin hydration with and without mannitol diuresis in refractory disseminated malignant melanoma: a South West Oncology Group Study. *Cancer Treat. Rep.*, **66**, 31.

CANADIAN MELANOMA GROUP (1984). Vinblastine, bleomycin and cisplatinum for the treatment of metastatic malignant melanoma. *J. Clin. Oncol.*, **2**, 131.

CAREY, R.W., ANDERSON, J.R., GREEN, M. & 3 others (1986). Treatment of metastatic malignant melanoma with vinblastine, dacarbazine and cisplatin: a report from the cancer and leukemia group B. *Cancer Treat. Rep.*, **70**, 329.

CHARY, K.K., HIGBY, D.J., HENDERSON, E.S. & 4 others (1977). Phase I study of high dose *cis*-dichlorodiammineplatinum (II) with forced diuresis. *Cancer Treat. Rep.*, **61**, 367.

COMIS, R.L. (1976). DTIC (NSC-45388) in malignant melanoma: a prospective. *Cancer Treat. Rep.*, **60**, 165.

COSTANZI, J.J. (1976). DTIC (NSC-45388) studies in the South-west Oncology Group. *Cancer Treat. Rep.*, **60**, 189.

CREAGAN, E.T., AHMANN, D.L., SCHUTT, A.J. & GREEN, S.J. (1981). Phase II study of the combination of vinblastine, bleomycin and cisplatin in advanced malignant melanoma. *Cancer Treat. Rep.*, **66**, 567.

EINHORN, L.H., BURGESS, M.A. & VALLEJOS, C. (1974). Prognostic correlations and response to treatment in advanced metastatic melanoma. *Cancer Res.*, **34**, 1995.

FRIEDMAN, M.A., KAUFMAN, D.A., WILLIAMS, J.E. & 7 others (1979). Combined DTIC and *cis*-dichlorodiammineplatinum (II) therapy for patients with disseminated melanoma. A Northern California Oncology Group Study. *Cancer Treat. Rep.*, **63**, 493.

HILL, G.J., KREMENTZ, E.T. & HILL, H.Z. (1984). Dimethyl triazeno imidazole carboxamide and combination therapy for melanoma. IV. Late results after complete response to chemotherapy (Central Oncology Group Protocols 7130, 7131 and 7131A). *Cancer*, **53**, 1299.

KARAKOUSIS, C.P., GETAZ, E.P., BJORNSSON, E.S. & 8 others (1979). *Cis*-dichlorodiaminneplatinum (II) and DTIC in malignant melanoma. *Cancer Treat. Rep.*, **63**, 2009.

LUIKART, S.D., KENNEALEY, G.T. & KIPKWOOD, J.M. (1984). Randomized phase III trial of vinblastine, bleomycin and *cis*-dichlorodiammine-platinum versus dacarbazine in malignant melanoma. *J. Clin. Oncol.*, **2**, 164.

MECHL, Z., NEKULOVA, M., SORKOVA, B. & 3 others (1983). The VBD regimen (vinblastine, bleomycin, cis-platinum) with high doses of cis-platinum in the therapy of advanced malignant melanoma. In *Proc. of the 13th International Congress of Chemotherapy*, p. 22.

NATHANSON, L., KAUFMAN, S.D. & CAREY, R.W. (1981). Vinblastine infusion, bleomycin and *cis*-dichlorodiammineplatinum chemotherapy in metastatic melanoma. *Cancer*, **48**, 1290.

ORATZ, R., SPEYER, J.L., GREEN, M. & 3 others (1987). Treatment of metastatic malignant melanoma with dacarbazine and cisplatin. *Cancer Treat. Rep.*, **71**, 877.

PRITCHARD, K.I., QUIRT, I.C., COWAN, D.H., OSOBA, D. & KUTAS, G.J. (1981). DTIC therapy in metastatic malignant melanoma: a simplified dose schedule. *Cancer Treat. Rep.*, **64**, 1123.

RETSAS, S., PEAT, I., ASHFORD, R. & 7 others (1980). Updated results of vindesine as a single agent in the therapy of advanced malignant melanoma. *Cancer Treat. Rev.*, **7** (suppl.), 87.

RETSAS, S., STOCHDALE, A. & NICOLL, J. (1985). Impact of chemotherapy on survival of patients with metastatic malignant melanoma: results of 240 patients treated at the Westminster Hospital. In *Proc. 3rd European Conference on Clinical Oncology*, p. 65.

WUSSOW, P.V., HARTMANN, F., BLOCK, B. & 3 others (1987). Treatment of advanced malignant melanoma with dacarbazine, vindesine and cisplatin (DVP). In *Proc. 4th European Conference on Clinical Oncology and Cancer Nursing*, p. 238.

WHO (1979). *Handbook for Reporting Results of Cancer Treatment.* World Health Organization: Geneva.

YORK. R., LAWSON, D.H. & McKAY, J. (1983). Treatment of metastatic malignant melanoma with vindesine, bleomycin by infusion and cis-platin. *Cancer*, **52**, 2220.


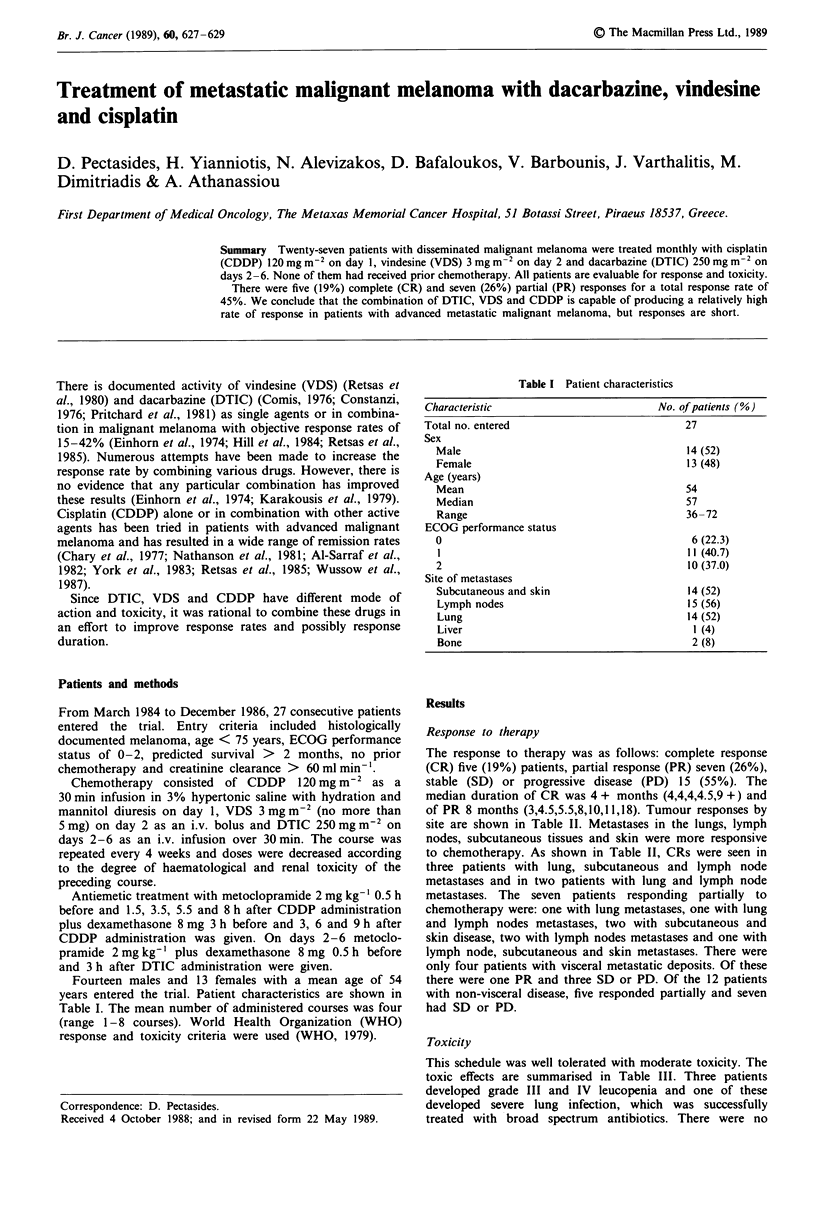

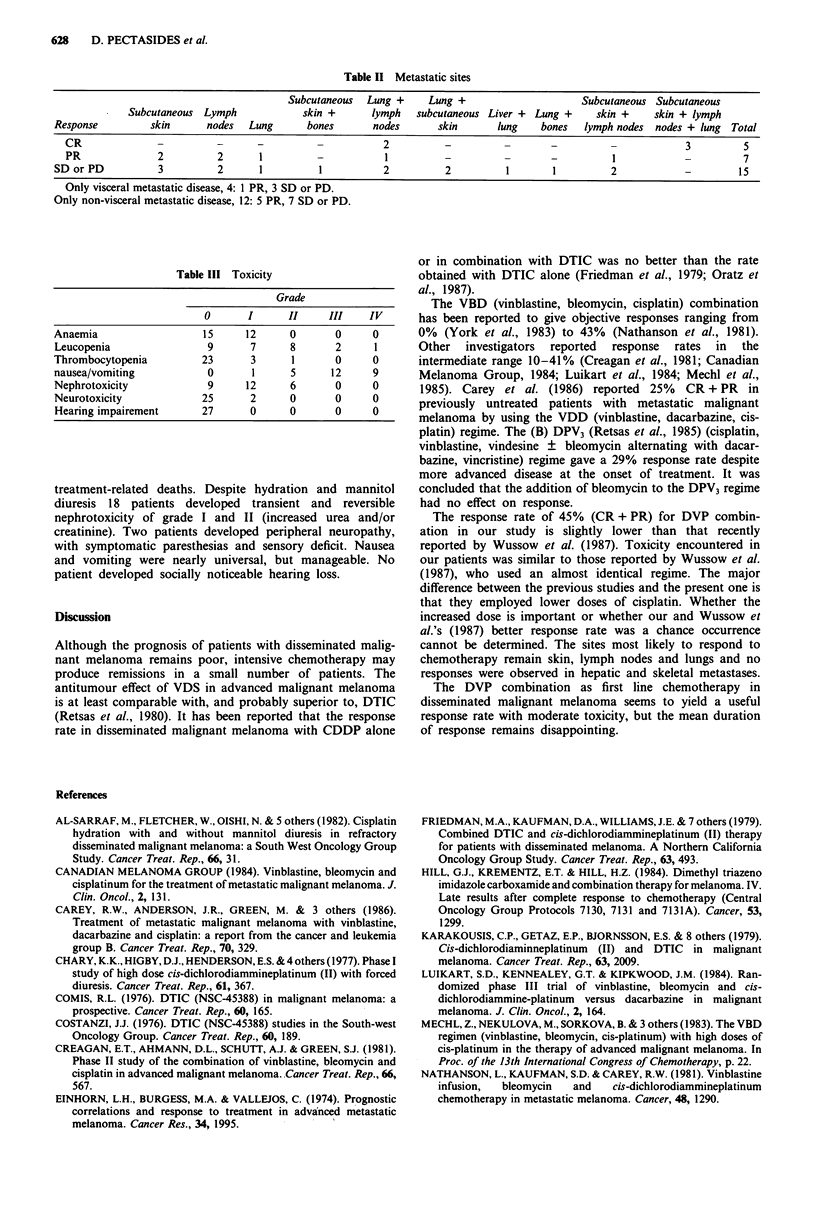

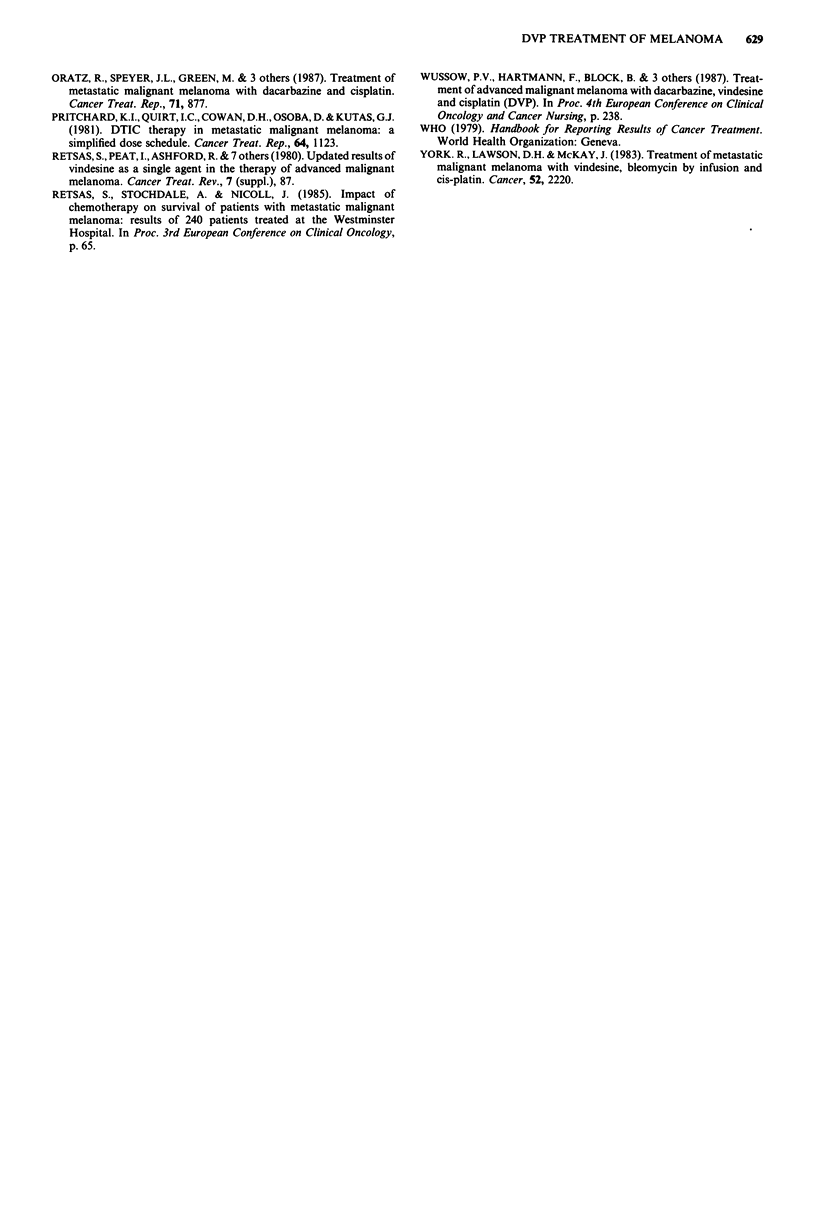


## References

[OCR_00242] Carey R. W., Anderson J. R., Green M., Ellison R. R., Nathanson L., Kennedy B. J. (1986). Treatment of metastatic malignant melanoma with vinblastine, dacarbazine, and cisplatin: a report from the Cancer and Leukemia Group B.. Cancer Treat Rep.

[OCR_00248] Chary K. K., Higby D. J., Henderson E. S., Swinerton K. D. (1977). Phase I study of high-dose cis-dichlorodiammineplatinum(II) with forced diuresis.. Cancer Treat Rep.

[OCR_00253] Comis R. L. (1976). DTIC (NSC-45388) in malignant melanoma: a perspective.. Cancer Treat Rep.

[OCR_00257] Costanzi J. J. (1976). DTIC (NSC-45388) studies in the southwest oncology group.. Cancer Treat Rep.

[OCR_00261] Creagan E. T., Ahmann D. L., Schutt A. J., Green S. J. (1982). Phase II study of the combination of vinblastine, bleomycin, and cisplatin in advanced malignant melanoma.. Cancer Treat Rep.

[OCR_00267] Einhorn L. H., Burgess M. A., Vallejos C., Bodey G. P., Gutterman J., Mavligit G., Hersh E. M., Luce J. K., Frei E., Freireich E. J. (1974). Prognostic correlations and response to treatment in advanced metastatic malignant melanoma.. Cancer Res.

[OCR_00272] Friedman M. A., Kaufman D. A., Williams J. E., Resser K. J., Rosenbaum E. H., Cohen R. J., Glassberg A. B., Blume M. R., Gershow J., Chan E. Y. (1979). Combined DTIC and cis-dichlorodiammineplatinum(II) therapy for patients with disseminated melanoma: a Northern California Oncology Group study.. Cancer Treat Rep.

[OCR_00278] Hill G. J., Krementz E. T., Hill H. Z. (1984). Dimethyl triazeno imidazole carboxamide and combination therapy for melanoma. IV. Late results after complete response to chemotherapy (Central Oncology Group protocols 7130, 7131, and 7131A).. Cancer.

[OCR_00285] Karakousis C. P., Getaz E. P., Bjornsson S., Henderson E. S., Irequi M., Martinez L., Ospina J., Cavins J., Preisler H., Holyoke E. (1979). cis-Dichlorodiammineplatinum(II) and DTIC in malignant melanoma.. Cancer Treat Rep.

[OCR_00290] Luikart S. D., Kennealey G. T., Kirkwood J. M. (1984). Randomized phase III trial of vinblastine, bleomycin, and cis-dichlorodiammine-platinum versus dacarbazine in malignant melanoma.. J Clin Oncol.

[OCR_00302] Nathanson L., Kaufman S. D., Carey R. W. (1981). Vinblastine, infusion, bleomycin, and cis-dichlorodiammine-platinum chemotherapy in metastatic melanoma.. Cancer.

[OCR_00309] Oratz R., Speyer J. L., Green M., Blum R., Wernz J. C., Muggia F. M. (1987). Treatment of metastatic malignant melanoma with dacarbazine and cisplatin.. Cancer Treat Rep.

[OCR_00314] Pritchard K. I., Quirt I. C., Cowan D. H., Osoba D., Kutas G. J. (1980). DTIC therapy in metastatic malignant melanoma: a simplified dose schedule.. Cancer Treat Rep.

[OCR_00341] York R. M., Lawson D. H., McKay J. (1983). Treatment of metastatic malignant melanoma with vinblastine, bleomycin by infusion and cisplatin.. Cancer.

